# Rising from the ashes: the return of The Climatic Physiology Group meeting

**DOI:** 10.1113/EP092641

**Published:** 2025-09-05

**Authors:** Josh Foster, Mike Tipton

**Affiliations:** ^1^ Centre for Human & Applied Physiological Sciences, School of Basic & Medical Biosciences, Faculty of Life Sciences & Medicine King's College London London UK; ^2^ Extreme Environments Laboratory University of Portsmouth Portsmouth UK

Between 1960 and 2003, there was an annual, 1‐day Climatic Physiology Group (CPG) meeting under the auspices of The Physiological Society. This meeting enabled scientists to discuss all aspects of their proposed, ongoing and completed work in a collegiate, supportive environment. Areas ranged from microgravity to hyperbaric physiology and everything in between. The attendees included senior scientists in the areas (names like John Bligh, Ken Collins, Frank Golden, Rainer Goldsmith, Romaine Hervey, David Elliott, John Ernsting, Bill Keating, David Kerslake, Jim Milledge, Griff Pugh and many others), as well as lots of PhD students and early career researchers. It was always well‐attended, motivational and incredibly helpful.

The aim of the CPG meeting was to facilitate the coming together of a wide range of post‐graduate and more senior scientists, and not just those with large travel budgets. Indeed, one of the main reasons for the meeting's success was the fact that it was deliberately low maintenance in terms of its organisation: no food, no teas or coffees, limited correspondence. As a result, it was usually free to attend. It was held over one day to avoid accommodation charges and was held in accessible places. The meeting rooms were provided for free, mostly in universities. As a result, people only had to find a bus or train fare, or parking spot, in order to attend. Presentations were generally short (5–10 min) to enable many to speak about their ideas or findings. The meeting stopped when too few people were researching extreme environmental physiology to warrant it.

But times change. There has been a significant growth in the number of laboratories researching extreme environmental physiology. Physiology is increasingly recognised as pivotal to global issues such as climate change, consequences of conflict, prolonged space travel, ageing and personalised medicine. Alongside this, fewer people have the funding to attend international meetings, and fewer still can afford to take all the researchers in their lab to such meetings. There is (rightly) an increasing reluctance, in the face of climate change, to fly around the globe, and stricter border controls make this increasingly difficult. Putting these factors together, it seems that now is a good time to resurrect the CPG meeting and the ethos which underpinned it: cheap, accessible, open to all. The meeting will be endorsed by The Physiological Society, and the CPG will work closely alongside other networks to advance the field of climatic physiology. The intention to do this was introduced at the recent Physiological Society meeting at Brunel University (‘Thermal physiology in health and disease: mechanisms and therapeutic applications’) and met with much enthusiasm and support. So, we are going to give it a go! We are going to try and offer an annual gathering that enables all of those working in a lab, from senior scientists to newly registered postgraduates, to attend a high‐quality scientific meeting, be informed and start creating new and wider networks.

One of the authors, J.F., has volunteered to take on the role of Secretary to the newly reformed CPG. The role of secretary will be held for an initial term of 3 years, with the possibility of renewal. The first meeting will take place in early September 2026 at King's College London, at the historic Great Hall, Strand Campus. It is strongly encouraged that presentations be given by PhD students and postdocs, since one of the main aims of the CPG is to increase accessibility to presentation experience and world‐class review from more experienced colleagues. However, a ‘finished article’ does not have to be presented; we encourage presentations on hypotheses and methodologies, up to near‐publication data. Talks are invited from all aspects of climatic physiology, including thermal stress, micro/hypergravity and hypo/hyperbaric physiology, encompassing laboratory, computational and field‐based experimentation. The core theme of the CPG is to network, celebrate science and progress this exciting and important area of physiology.

We look forward to welcoming colleagues with an interest in climatic physiology to King's College London as we renew this excellent tradition and advance the field through open, collegiate exchange. More specific details will be released in the coming months.

## AUTHOR CONTRIBUTIONS

Both authors contributed to the conception or design of the work, acquisition, analysis or interpretation of data for the work, drafting the work or revising it critically for important intellectual content. Both authors approved the final version of the manuscript, agree to be accountable for all aspects of the work in ensuring that questions related to the accuracy or integrity of any part of the work are appropriately investigated and resolved; and all persons designated as authors qualify for authorship, and all those who qualify for authorship are listed.

## CONFLICT OF INTEREST

None declared.

## FUNDING INFORMATION

No funding was received for this work.

